# Effects of Shenfu Injection and Its Main Components on the Contraction of Isolated Rat Thoracic Aortic Rings

**DOI:** 10.1371/journal.pone.0078026

**Published:** 2013-10-30

**Authors:** Jinqiang Zhu, Liyuan Kang, Qiaofeng Ye, Guanwei Fan, Yubin Liang, Chen Yan, John Orgah

**Affiliations:** 1 Institute of Traditional Chinese Medicine, Tianjin Key Laboratory of Chinese Medical Pharmacology, Tianjin University of Traditional Chinese Medicine, Tianjin, People’s Republic of China; 2 Research and Development Center of TCM, Tianjin International Joint Academy of Biotechnology and Medicine, Tianjin, People’s Republic of China; Thomas Jefferson University, United States of America

## Abstract

**Background and Purpose:**

*Shenfu injection* (SFI), derived from the ancient traditional Chinese medicine (Red Radix Ginseng and Radix Aconitum Carmichaeli), has been widely used in the clinical for the treatment of cardiovascular diseases for more than 20 years. The present study aims to investigate the effects of SFI and its main components on the contraction of isolated rat thoracic aorta rings and the potential mechanisms of this action.

**Methodology/Principal Findings:**

The isolated rat thoracic aorta rings were initially treated with different concentrations of SFI, *Hongshen injection* (HSI, mainly containing ginsenoside) or *Fupian injection* (FPI, mainly containing aconite total alkaloids) separately. The control group was added an equal volume Krebs-Henseleit (K-H) solution. All three injections exhibited no obviously effects on the basal tension of the rings in the resting state. However, in the isolated thoracic aorta rings with intact endothelium, when the rings were first induced by 60 mM potassium chloride (KCl) or 1 µM norepinephrine (NE) to the maximal contraction and then treated with above injections, SFI and HSI significantly inhibited the vasoconstriction induced by KCl or NE. In addition, FPI has a tendency to inhibit KCl-induced vasoconstriction and facilitate NE-induced vasoconstriction, but no significant difference. None of them showed obvious effect on the endothelium denuded vessels. Moreover, this procedure was repeated after pre-incubation of nitric oxide synthase (NOS) inhibitor N^G^-nitro- L-arginine methyl ester (L-NAME), which suppressed the vasorelaxation effect of SFI and HSI.

**Conclusions and Implications:**

These results demonstrate that both SFI and HSI caused an apparent thoracic aorta relaxation by endothelium-dependent manner, which was associated with eNOS system, while FPI had no detectable vasodilator effect. This suggested that the ginsenoside from red Radix Ginseng may be the main active ingredient of SFI’s vasodilator effect.

## Introduction

*Shenfu injection* (SFI) is originated from the ancient prescriptions *Shenfu* formula, which is composed of red Radix Ginseng (*Hongshen*) and Radix Aconitum Carmichaeli (*Fupian*). Its main active components include ginsenoside and aconite total alkaloids. Numerous studies have shown that SFI possesses many pharmacologic actions, including dilating coronary artery, improving microcirculation, increasing blood pressure and strengthening myocardial contractility, thus it is commonly used to treat congestive heart failure, shock, arrhythmia, acute myocardial infarction, pulmonary heart disease, viral myocarditis, dilated cardiomyopathy, and so on [1]–[10]. Previous studies have shown that ginsenoside, the main active ingredient in SFI, had a bi-directional regulation of blood pressure [11], [12]. Another component of SFI, monkshood aconite, had excitatory effects on both α and β-adrenergic receptor. The binding affinity of monkshood aconite to β-adrenergic receptor was similar to isoproterenol [13]. The clinical trials have demonstrated that SFI, had a bi-directional adjustment on blood pressure thereby to maintain blood pressure in a normal level [14], [15], but its possible mechanism is still unknown. Currently, most studies had paid attention to some mechanisms of SFI to treat the cardiovascular diseases, such as increasing vascular perfusion, improving hemodynamics and hemorheology, enhancing the ATP activity in myocardial cell membrane, regulating cardiac myocyte electrophysiology effect, sodium ion channels and cytokines, and so on [16]. However, there are few systematic studies on its vascular protective effects and related mechanisms of action. Especially, the interaction between the two main active components, ginsenoside and aconite total alkaloids, have not been discussed yet. Indeed, it is very necessary to explore what is the main active ingredient of SFI’s effects. In order to explore the action characteristics and the potential mechanisms of SFI, *Hongshen injection* (HSI) and *Fupian injection* (FPI), which contain its main components ginsenoside and aconite total alkaloids respectively, we used isolated rat thoracic aortic rings in the present study to investigate the effects of the three injections on the vasoconstriction induced by KCl or NE. We also examined the influence of the NO synthase inhibitor L-NAME on the vasodilator of SFI and HSI.

## Materials and Methods

### Animal

Male *Wistar* rats (weighing 250–300 g, specific pathogen free) were obtained from Beijing HFK Bioscience. Co., Ltd (Permit No.: SCXK (Jing 2009-0004)). This study was carried out in strict accordance with the recommendations in the Guidance Suggestions for the Care and Use of Laboratory Animals issued by the Ministry of Science and Technology of China. The protocol was approved by the the Laboratory Animal Ethics Committee of Tianjin University of Traditional Chinese Medicine (Permit Number: LAEC2013002).

### Regents and Drugs Preparation

Norepinephrine (NE), Acetyleholine (Ach), L-NAME were purchased from Sigma (St. Louis, MO, U.S.A.), NaCl, KCl, KH_2_PO_4_, MgSO_4_·7H_2_O, CaC1_2_, NaHCO_3_ and Glucose from Tianjin Chemical Reagent Factory (Tianjin, China). Other chemicals were used in analytical grade.

SFI, HSI and FPI were provided by Yaan Sanjiu Pharmaceutical Co., Ltd. (NO.110804, Yaan, China), SFI contains 1 mg/ml red Radix Ginseng (*Hongshen*) and 2 mg/ml Radix Aconitum Carmichaeli (*Fupian*), HSI and FPI contain 1 mg/ml red Radix Ginseng and 2 mg/ml Radix Aconitum Carmichaeli respectively.

Drugs were prepared as working procedure [17]: The red Ginseng Radix was crushed, extracted with ethanol 4 times under reflux (2 h/time), after soak in ethanol for 2 hours, then combined the ethanol solution concentrated in a rotator evaporator in vacuo to give red Ginseng Radix extract. 22 compounds including 12 kinds major ginsenosides and 10 kinds rare ginsenosides were separated from the extracts of red Ginseng Radix and identified as ginsenoside Rg_1_, ginsenoside Re, ginsenoside Rf, ginsenoside Rg_2_, ginsenoside Rb_1_, ginsenoside Rc, ginsenoside Rb_2_, ginsenoside Rb_3_, ginsenoside Rd, ginsenoside F_1_, ginsenoside F_3_ and ginsenoside F_5_, ginsenoside Rk_1_, ginsenoside Rg_5_, ginsenoside Rk_3_, ginsenoside Rh_4_, ginsenoside Rg_6_, ginsenoside F_4_, ginsenoside Rg_3_, ginsenoside 20(R)-Rg_3_, ginsenoside Rh1and ginsenoside 20(R)-Rh_1_. Different production batches of 20 kinds ginseng saponins content were range from 85–119 (Rg_1_), 58–89 (Re), 28–31 (Rf), 13–15 (Rg_2_), 198–227 (Rb_1_), 97–114 (Rc), 50–64 (Rb_2_), 8–14 (Rb_3_), 3–6 (F_1_), 36–49 (Rd), 27–42 (Rh_1_), 14–25 (20 (R)-Rh_1_), 3–6 (Rg6), 4–8 (F4), 8–11 (Rk_3_), 6–11 (Rh_4_), 21–36 (Rg_3_), 16–30 (20 (R)-Rg_3_), 8–15 (Rk_1_), 12–20 (Rg_5_) µg·ml^−1^.Aconite was extracted with water twice (2 h/time), after soakin 1% HCl water solution for 2 hours. The combined solution was concentrated in vacuo, after it cool down, add appropriate amount of ethanol, then concentrated in a rotator evaporator to generate aconite extract. Six compounds were separated and identified from the extracts of Aconiti, they are benzoyldeoxyaconitine (BD), benzoylhypaconitine (BH), benzoylaconitine (BA), benzoylmesaconitine (BM), aconine and hypaconine. 4 kinds alkaloids content from different production batches of Shenfu injection ranged from 0.2–1.2 (BM), 0.2–0.3 (BA), 0.1–0.7 (BH) and 0.04–0.1 (BD) µg·ml^−1^.

### Preparation of Isolated Rat Thoracic Aortic Rings

Male *Wistar* rats (250–300 g) were exsanguinated after decapitation. The thoracic aorta was immediately isolated and immersed in Krebs-Henseleit (K-H) solution (the composition of NaCl 118 mM, KCl 4.75 mM, MgSO_4_·7H_2_O 1.2 mM, KH_2_PO_4_ 1.2 mM, CaCl_2_ 2.5 mM, NaHCO_3_ 25 mM and D-glucose 11 mM) [18]. After careful removal of adherent tissues, four to eight 3 mm long rings were cut. Care was taken not to damage the inner surface of the blood vessel, or denuded aorta rings were prepared by mechanical method with cotton pipe-cleaners. The rings were suspended in the organ bath (Radnoti, AD Instruments Pty Ltd., Australia) between two parallel stainless steel hooks. One hook was fixed, while the other was connected to a force transducer (AD Instruments Pty Ltd., Australia) for the measurement of isometric tension. The organ bath contained 10 ml K-H solution with pH of 7.3–7.4 at 37.0°C, and bubbled with 95% O_2_ and 5% CO_2_ to keep blood vessels alive. Isometric tension change was measured with the force-displacement transducer and recorded by PowerLab 8/30 bio-signal recording system (AD Instruments Pty Ltd, Australia). The aorta rings were stretched progressively to a basal tension of 2.0 g and allowed to equilibrate for at least 1.5 h, during this period of time the bath solution was replaced with pre-warmed and oxygenated K-H solution every 15 min and the tension was readjusted to 2.0 g. To standardize the experiments after stabilization at the beginning of the experiments the rings were repeatedly contracted with 60 mM KCl till the difference of contraction plateau remained within 15% between two consecutive contractions, after which they were rinsed with pre-warmed and oxygenated K-H solution three times until muscle tension returned to the basal level.

### Testing the Endothelium Integrity

**Figure 1:**
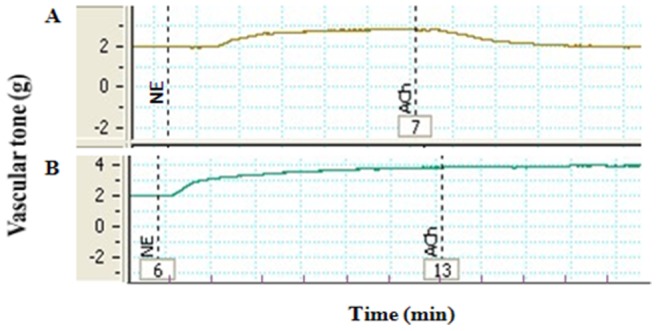
Testing the endothelium integrity. The aorta rings were pre-treated with 1 µM NE to achieve plateau phase, then 10 µM Ach was added to induce the vasodilation. A: the relaxation extent>80% *vs* plateau phase; B: the relaxation extent<30% *vs* plateau phase.

The aorta rings were pre-treated with 1 µM NE to achieve plateau phase, then 10 µM ACh was added to induce the vasodilation. Compared with the maximum contraction extent induced by 1 µM NE, if relaxation extent achieved is more than 80%, the endothelium would be regarded as intact and functional, while less than 30%, the aorta rings would be regarded as completely denuded endothelium (Figure 1).

### Measurement of Vascular Relaxation

Contractility of vascular rings was measured isometrically. Once a sustained contraction plateau with 60 mM KCl or 1 µM NE was achieved, SFI, HSI or FPI (0.l–10 ml/L) were added cumulatively with an interval of 5 min on rings with or without functional endothelium into the organ bath. In order to investigate the involvement of the endothelium NO in vasorelaxation to SFI and HSI, some tissues were exposed to 1 µM L-NAME for 20 min prior to application of NE to blunt the endothelial function by inhibiting NO synthesis after repeated washout and subsequent equilibration for about 45 min.

### Statistical Analysis

Relaxant responses are given as percentage contraction relative to pre-contraction levels to KCl or NE. All values are represented as mean ± S.E.M. of n experiments. Statistical significance was estimated by Student’s *t*-test for unpaired observation between two groups. *P*-values less than 0.05 were regarded as significant. In all experiments, n is the number of rats from which vessel segment were obtained.

## Results

### SFI, HSI and FPI have No Significant Effect on the Basal Tension of Normal Blood Vessels

**Figure 2:**
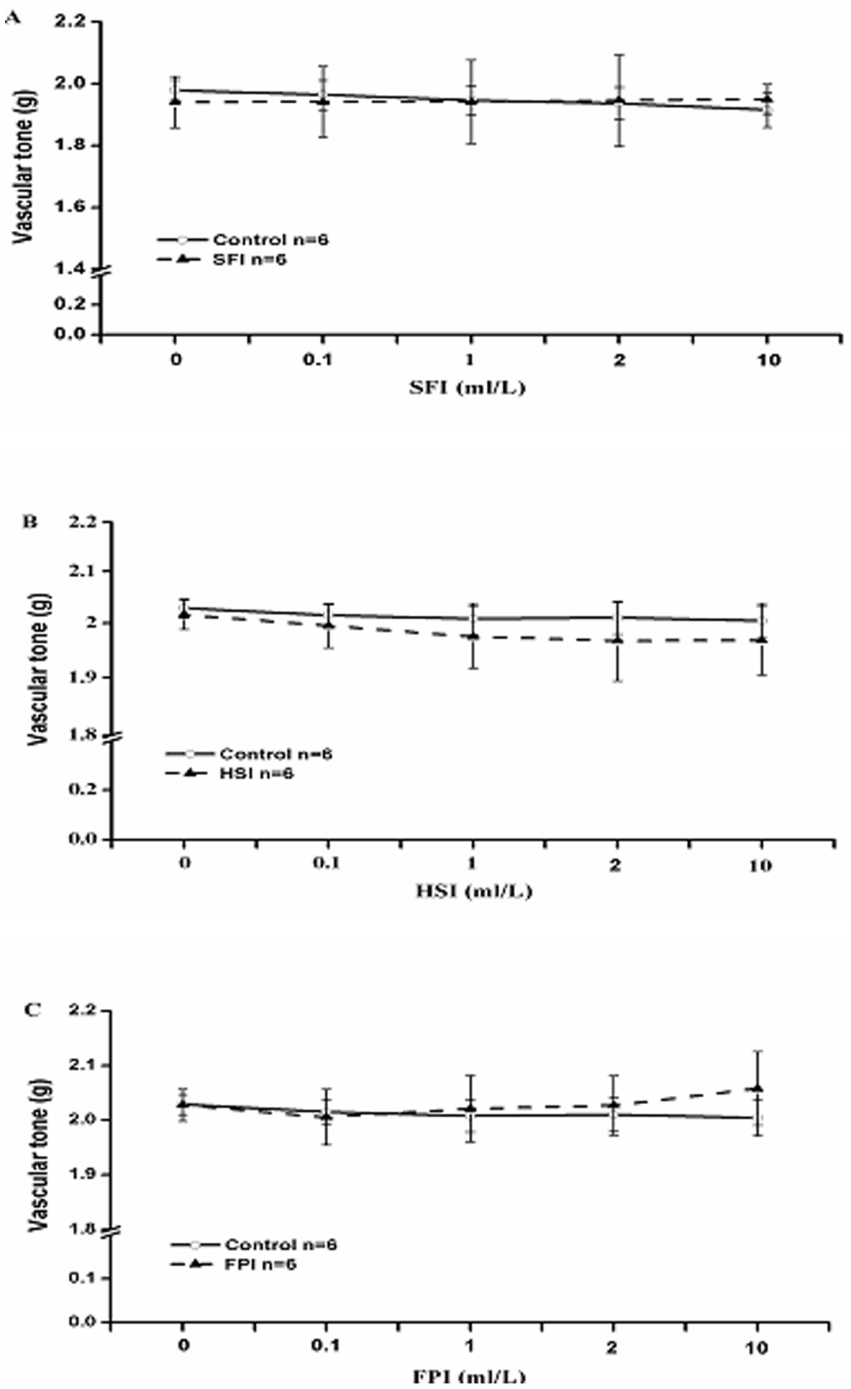
After being progressively stretched to a basal tension of 2.0 g and equilibrated for at least 90 min, different concentrations (0.l-10 ml/L) of SFI (A), HSI (B) and FPI(C) were added cumulatively. They had no significant effect on the basal tension of normal blood vessels.

To observe whether these three injections have effects on the basal tension of normal blood vessels, some normal aorta rings were treated with different concentration of them, and the control group was added an equal volume K-H solution, after being stretched progressively to a basal tension of 2.0 g and equilibrated for at least 90 mins. Our data demonstrated that SFI, HSI and FPI had no significant effect on the basal tension of normal blood vessels (Figure 2).

### Effects of SFI, HSI and FPI on KCl-induced Vasoconstriction with Denuded and Intact Endothelium

**Figure 3:**
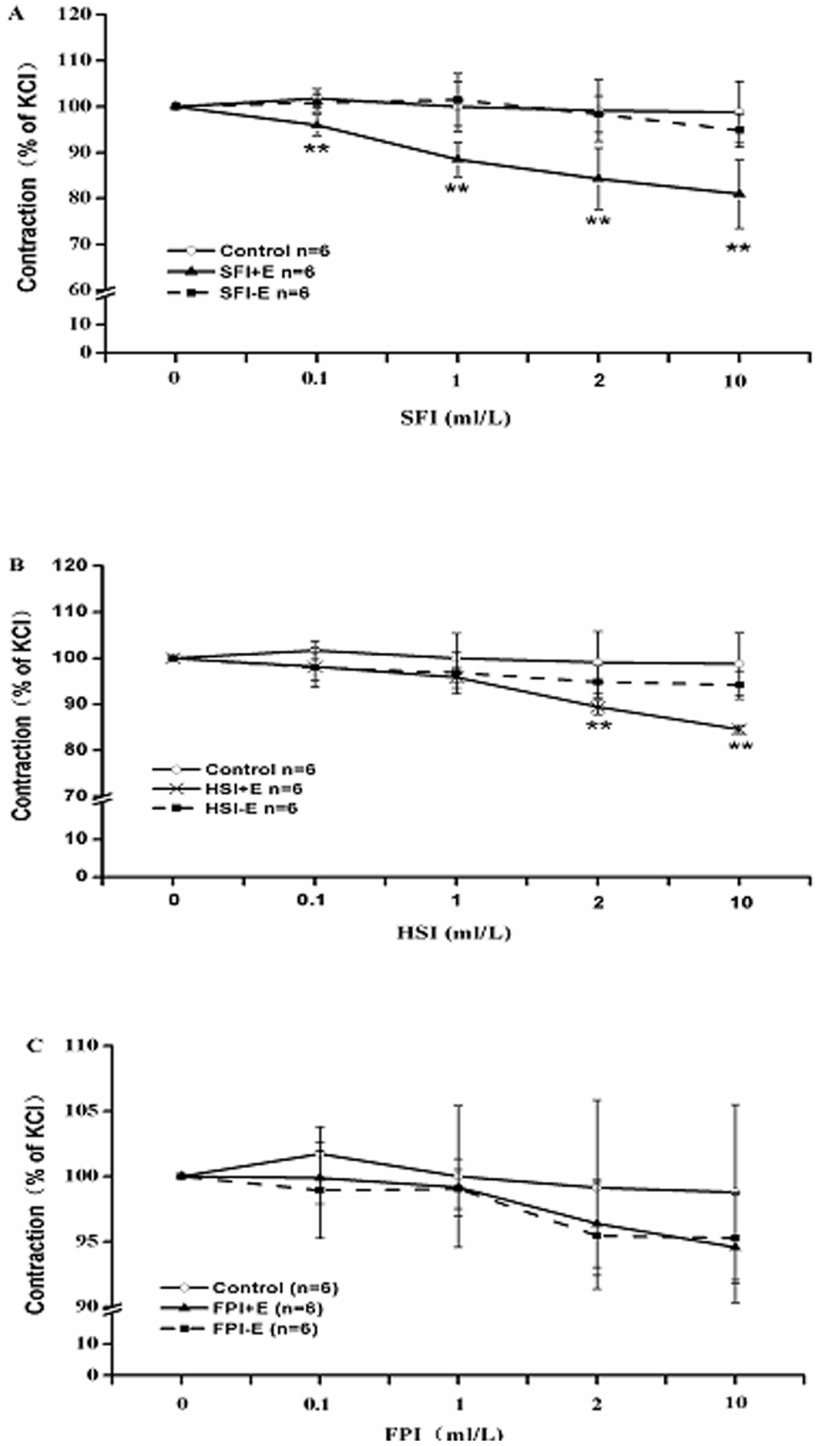
The inhibition of KCl (60 mM)-pre-contracted rat thoracic aorta ring with endothelium denuded (−E) and with intact endothelium (+E) in response to cumulative addition of SFI, HSI and FPI. Each dose of SFI can significantly inhibit KCl-induced contraction of aorta rings with intact endothelium, while had no significant effect on the contraction of aorta rings with denuded endothelium (A); 2 ml/L and 10 ml/L of HSI had a similar role as SFI (B); FPI had no significant effect on KCl-induced contraction of thoracic aorta ring with denuded and intact endothelium (C). **P<0.01 *vs* Control (the control group was added an equal volume K-H solution).

The depolarization in vascular smooth muscle cell membrane can be caused by the high concentrations of K^+^, which can also open the voltage-dependent calcium channels (VDC), then cytosolic free Ca^2+^ will increase as a result of Ca^2+^ influx, which causes smooth muscle contraction. Therefore, we treated the vascular rings with 60 mM KCl to induce vasoconstriction, then added SFI, HSI or FPI (0.l ml/L, 1 ml/L, 2 ml/L, 10 ml/L) accumulatively to detect their effects on KCl-induced vasocontraction. As our results showed, each dose of SFI can significantly inhibit KCl-induced aorta rings contraction with intact endothelium, while had no obvious effect on the contraction of ones without endothelium (Figure 3A); 2 ml/L and 10 ml/L of HSI had a similar role as SFI (Figure 3B), which demonstrated that both of them had endothelium-dependent inhibited KCl-induced vasocontraction. Otherwise, FPI had no significant effect on KCl-induced contraction of thoracic aorta ring with denuded and intact endothelium (Figure 3C).

### Effects of SFI, HSI and FPI on NE-induced Vasoconstriction with Denuded and Intact Endothelium

**Figure 4:**
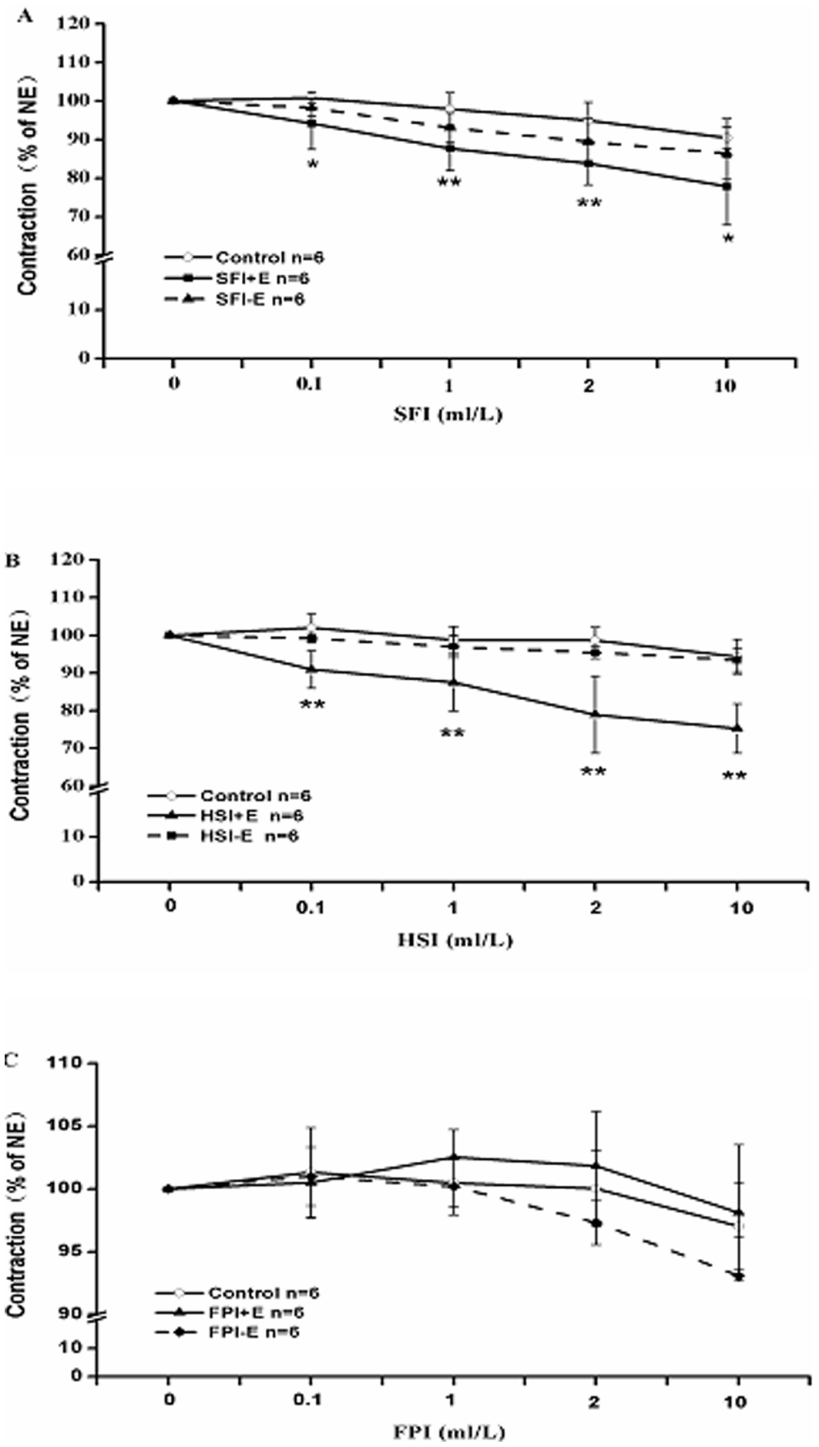
The inhibition of NE (1 µM)-pre-contracted rat thoracic aorta rings with endothelium denuded (−E) and with intact endothelium (+E) in response to cumulative addition of SFI, HSI and FPI. SFI can markedly suppress the intact endothelium ring contraction induced by NE, while had no obviously influence on the contraction of endothelium-denuded aorta rings (A); HSI also had a similar role as SFI (B); FPI also had no significant effect on NE-induced contraction of thoracic aorta ring with denuded and intact endothelium (C). *P<0.05, **P<0.01 *vs* Control (the control group was added an equal volume K-H solution).

After interaction with α receptor, NE can open receptor-operated calcium channels (ROC), then Ca^2+^ influx causes smooth muscle contraction. Thus, we used 1 µM NE to trigger vasoconstriction, and then added 0.l ml/L, 1 ml/L, 2 ml/L, 10 ml/L SFI, HSI or FPI cumulatively in order to detected their effects on NE-pre-contracted rat thoracic aorta rings. Our data indicated that SFI can markedly suppress the intact endothelium rings contraction induced by NE, while had no obvious influence on the contraction of endothelium-denuded aorta rings (Figure 4A); HSI had a similar action (Figure 4B), suggesting that they were able to endothelium-dependently inhibit NE-induced vasocontraction. In addition, FPI also had no significant effect on NE-induced contraction of thoracic aorta ring with denuded and intact endothelium (Figure 4C).

### L-NAME Suppresses the Vasorelaxation of SFI and HSI

**Figure 5:**
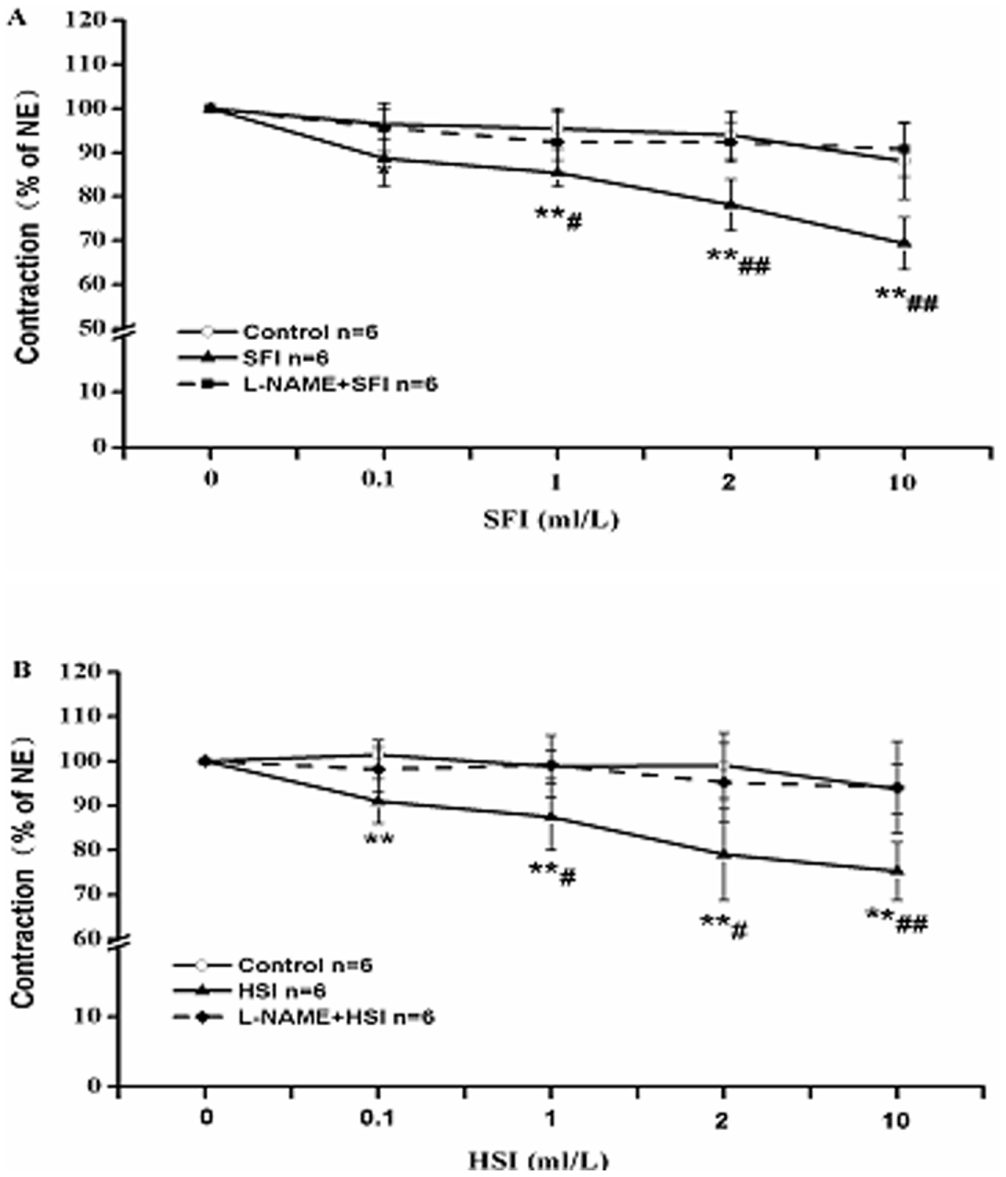
The inhibition of NE (1 µM)-pre-contracted rat thoracic aorta rings with intact endothelium in response to cumulative addition of SFI and HSI in the presence and absence of 1 µM L-NAME. L-NAME can obviously restrain the vasorelaxation of SFI (A) and HSI (B). **P<0.01 *vs* Control (the control group was added an equal volume K-H solution); ^#^ *P*<0.05, ^##^ *P*<0.01 *vs* SFI or HSI.

It is well known that NO is a major endothelium-derived relaxing factor, which is generated from L-arginine and catalyzed by endothelial NO synthase (eNOS) in vascular endothelium. To detect the role of NO in the vasorelaxation induced by SFI and HSI, we pre-treated the thoracic aorta rings with 1 µM eNOS inhibitor L-NAME. It found that L-NAME can almost completely abolish the relaxant effects induced by SFI and HSI (figure 5).

### The Difference of the Three Injections on Vasorelaxation

As mentioned above, HSI can significantly inhibit KCl-induced contraction of aorta rings with intact endothelium by a decline of 3.55%, 4.10%, 9.71%, 14.20%; FPI emerged an inhibition trend with 1.84%, 0.83%, 2.74%, 4.23%; SFI also can significantly inhibit this contraction declined to 5.76%, 11.49%, 14.82%, 17.80% respectively; SFI exhibited stronger inhibitory effect than HSI. Moreover, HSI can markedly suppress vasoconstriction induced by NE by a decline of 11.05%, 11.35%, 19.67%, 19.08%; FPI appeared a promotion trend of 0.84%, −2.07%, −1.80%, −1.09%; SFI also can significantly restrain it declined by 6.70%, 10.21%, 11.16%, 12.57% respectively, and the role of HSI dose better than that of SFI (Table 1).

**Table 1:** Decline of HSI, FPI and SFI inhibiting KCl, NE-induced vasocontraction (%).

	KCl-induced vasocontraction				NE-induced vasocontraction			
	0.1 ml/L	1 ml/L	2 ml/L	10 ml/L	0.1 ml/L	1 ml/L	2 ml/L	10 ml/L
**HSI**	3.55	4.10	9.71	14.20	11.05	11.35	19.67	19.08
**FPI**	1.84	0.83	2.74	4.23	0.84	−2.07	−1.80	−1.09
**SFI**	5.76	11.49	14.82	17.80	6.70	10.21	11.16	12.57

## Discussion

In China, SFI has been widely used in the clinical for treatment of cardiovascular diseases because of its significant cardiovascular protective effects. The previous results indicated that SFI had a protective effect on blood vessel endothelium, which can stabilize and increase the mean arterial pressure of rabbit with a cardiac arrest caused by hypoxia, also can shorten the recovery time of mean arterial pressure and attenuate postresuscitation myocardial dysfunction through beneficial effects on energy metabolism and remarkable antioxidant capacity [19]–[21]. But there is scarcely research about its effect on vasomotor function. Therefore, in this study we investigated the effects of SFI on KCl-induced or NE-induced constriction of the thoracic aorta rings with denuded and intact endothelium. The results show that SFI can significantly induce endothelium-dependent relaxation (Figure 3A, 4A), which may improve microcirculation and form beneficial effect on cardiovascular diseases. The results also suggest that the effects of SFI on hypertension and pressure stabilize are relevant to its regulation of vascular tone, which will provide a theoretical basis for its further research and clinical applications.

In vascular endothelial cells, eNOS catalyzes L-arginine to generate NO, which plays an important role in the regulation of vascular relaxation. NO can soon diffuse to the vascular smooth muscle cell once formed, activate the guanylate cyclase and promote to generate cGMP, which activates cGMP-dependent protein kinase (PKG), and affects the exchange of Na^+^ and Ca^2+^, finally relaxes the vascular smooth muscle and blood vessel [22]. In order to study the effect of eNOS on the vasorelaxation of SFI, we pre-treated some thoracic aorta rings with NOS inhibitor L-NAME, then added NE and different dosages of SFI. Our data demonstrate that the vasorelaxation of SFI is associated with NO generated in endothelial cells (Figure 5A). Whether, SFI works through influence on the eNOS activity and the formation of NO requires further research.

As a Chinese herbal formula, SFI is characterized by multi-component, multi-target and multi-effect. Numerous researches have demonstrated that it possesses complex pharmacologic actions. Zheng CD et al. [23] found it has cardioprotection against myocardial ischemia/reperfusion (I/R) injury in open heart surgery via significantly improving the systolic blood pressure (SBP), mean blood pressure (MBP), diastolic blood pressure (DBP) values, the recovering rate of spontaneous heart beat, heart rate (HR), electrocardiographic (ECG) ST segment, atrioventricular block, ventricular arrhythmia and superoxide dismutase (SOD), and significantly decreasing the serum concentration of cardiac troponin I (cTnI), malondialdehyde (MDA). Zheng SY et al. [24] also reported that SFI can remarkablely inhibit the rise in MDA and lactate dehydrogenase (LDH) and as well as creatine kinase (CK), increase SOD activity, reduce the size of infarction, improve the pathologic changes of myocardium and inhibit NF-κB activity leading to down-regulation of pro-inflammatory cytokine (TNF-α and IL-6) expression during I/R [25]. In addition, it also can modulate the protein espression of Bcl-2 and Bax genes and reduce Caspase-3 activity as to inhibiting apoptosis, meanwhile inhibit endo-myocardial cell calcium releasing of sarcoplasmic reticuium to avoid calcium overload induced by adriamycin, to achieve the effect of protection myocard [26]–[28]. On the contrary, it can promote L-type calcium channels in the normal myocardial cell membrane opening, which cause calcium influx, leading to strengthening myocardial contractility [29]. Furthermore, SFI plays a potent role in improving hemorheology, preventing impaired myocardial β-adrenergic receptor (AR) signaling to attenuate post-resuscitation myocardial dysfunction after cardiopulmonary resuscitation (CPR), mitigating lung injury through reducing the levels of IL-4, increasing levels of IFN-γ, and inducing T-bet mRNA upregulation and GATA-3 mRNA downregulation [30]–[32], and so on. Thus study on its material basis has a great significance to investigate its pharmacological mechanism. As the compound composition is not clear, it is difficult to accurately explain the pharmacodynamic material basis and the action mechanism exclusively using Chinese materia medica formula to intervene in the pathological animal models. While application of Chinese materia medica monomer for the mechanism research cannot in line with the thinking of Chinese materia medica compatibility. Chinese materia medica components compatibility study may find the balance of the two study modes, make up for the lack of them to some extent, better clarify the connotation of Chinese medicines compatibility, and promote the modernization of traditional Chinese medicine (TCM) [33], [34]. To explore the main active ingredient and the potential mechanism of SFI’s vasorelaxation effect, this experiment investigated effects of HSI containing ginsenoside and FPI containing aconite alkaloids on isolated rat thoracic aortic ring contraction induced by KCl or NE. The results showed that HSI had significant endothelium-dependent relaxation of the thoracic aorta (Figure 3B, 4B), and this action was related with NO releasing in vascular endothelial cells (Figure 5B), which was consistent with Kang SY’s result [35], [36]. Meanwhile, it suggested that it is probably ginsenoside, the main active ingredient of SFI, that contributes to SFI’s vasodilator effect. Moreover, compared with HSI, inhibition of SFI to KCl-induced vasoconstriction was stronger than HSI, while the latter was stronger than the former when they were used to inhibit NE-induced vasoconstriction. FPI had no apparent vasodilator. That is possibly because the thoracic aorta is not the action target of FPI, which suggest that the effect levels of different components on these two kinds of calcium channels are different, since KCl and NE act on the voltage-dependent calcium channels (VDC) and receptor-operated calcium channels (ROC) respectively.

**Figure 6:**
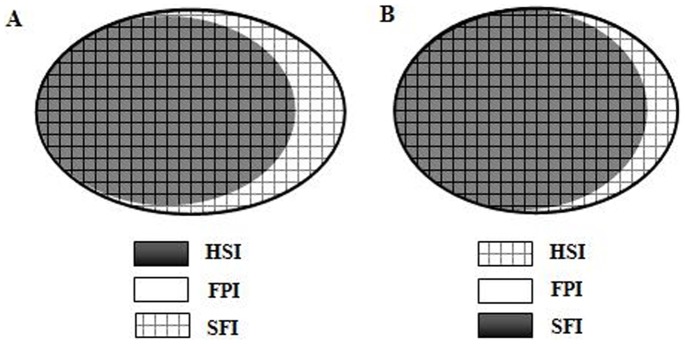
The inhibition of SFI to KCl-induced vasoconstriction was better than that of HSI, and FPI had an inhibition trend to it (A). Moreover, the supression of HSI to NE-induced vasoconstriction was senior compared with SFI, and FPI appeared an inhibition tendency (B). The ellipse size is on behalf of the contraction decline of these three injections.

As shown in Table 1, when the vascular endothelium is intact, for KCl-induced vasoconstriction, the relaxation effect of HSI is worse than that of SFI, which may be because FPI has a trend to vasorelaxation, and the effect of SFI is synergy between HSI and FPI; for NE-induced vasoconstriction, the relaxation effect of HSI is better than that of SFI, which may be because FPI has a trend to vasoconstriction, and the effect of SFI is antagonism between HSI and FPI. It also suggested that the effect levels of different components in SFI on these two kinds of calcium channels (VDC and ROC) are different. Thus enhancing weak effect and inhibiting strong effect maybe one of the compatibility rules of traditional Chinese medicine (Figure 6), but the exact mechanism remains to be further studied.
